# Therapeutic Implications of Ferroptosis in Renal Fibrosis

**DOI:** 10.3389/fmolb.2022.890766

**Published:** 2022-05-17

**Authors:** Yao Zhang, Yanhua Mou, Jianjian Zhang, Chuanjian Suo, Hai Zhou, Min Gu, Zengjun Wang, Ruoyun Tan

**Affiliations:** ^1^ Department of Urology, The First Affiliated Hospital of Nanjing Medical University, Nanjing, China; ^2^ Department of Oncology, Xiangyang Central Hospital, Affiliated Hospital of Hubei University of Arts and Science, Xiangyang, China; ^3^ Department of Urology, The Second Affiliated Hospital of Nanjing Medical University, Nanjing, China

**Keywords:** renal fibrosis, ferroptosis, iron homeostasis, lipid peroxidation, programmed cell death

## Abstract

Renal fibrosis is a common feature of chronic kidney disease (CKD), and can lead to the destruction of normal renal structure and loss of kidney function. Little progress has been made in reversing fibrosis in recent years. Ferroptosis is more immunogenic than apoptosis due to the release and activation of damage-related molecular patterns (DAMPs) signals. In this paper, the relationship between renal fibrosis and ferroptosis was reviewed from the perspective of iron metabolism and lipid peroxidation, and some pharmaceuticals or chemicals associated with both ferroptosis and renal fibrosis were summarized. Other programmed cell death and ferroptosis in renal fibrosis were also firstly reviewed for comparison and further investigation.

## 1 Introduction

Chronic kidney disease (CKD) is an important public health concern causing a high economic cost to the health systems, with an overall prevalence of more than 10% (Hill et al., 2016). Progressive CKD often results in end-stage renal failure and the only options are dialysis or kidney transplantation. Renal fibrosis is a common characteristic of CKD independent of the underlying etiology, leading to the destruction of normal kidney structure and loss of renal function. Renal fibrosis, one of the common characteristics of CKD, includes tubulointerstitial fibrosis and glomerulosclerosis, regardless of the underlying etiology, is synthesized from an imbalance between excessive synthesis and reduced breakdown of the extracellular matrix, and it may be due to normal wound healing response become uncontrolled, with an uncontrolled inflammatory response and myofibroblast proliferation, eventually leading to destruction of normal kidney structure and loss of renal function (el Nahas et al., 1997). Currently, therapies against renal fibrosis exhibit limited effectiveness, and little progress has been made in reversing fibrosis in recent years.

In 2003, Sonam Dolma et al. discovered a new form of cell death induced by erastin without nuclear morphological changes, DNA fragmentation, and caspase-3 activation, which could not be reversed by caspase inhibitors (Dolma et al., 2003) but could be inhibited by iron chelator (Yang and Stockwell, 2008). Subsequently, Dixon named this cell death “ferroptosis” in 2012 (Dixon et al., 2012), which is characterized by lipid peroxidation-mediated membrane damage, leading to iron-dependent regulated necrosis (Stockwell et al., 2017). Different from autophagy, necrosis, pyroptosis, and apoptosis, ferroptosis morphologically exhibits normal nuclei and shrinking mitochondria with condensed mitochondrial membrane, diminished or abolished mitochondria cristae, and ruptured outer mitochondrial membrane (Yagoda et al., 2007; Cao and Dixon, 2016; Latunde-Dada, 2017). The connection between necrotic cell death and long-term renal fibrosis may be dominated by the inflammatory infiltration, which could be defined by the type of necrotic death (Tang et al., 2020). Although our knowledge about the role of ferroptosis in renal fibrosis is still in its infancy, the cytotoxicity of iron and lipid peroxidation has been known since the 1900s and 1950s, respectively (Moore and Hawkes, 1908; Bieri, 1959). Before the proposing of ferroptosis, many studies confirmed that the ferroptosis-related indicators, including GPXs, iron, and lipid peroxides are participated in renal fibrosis (Sponsel et al., 1996; Ueda et al., 1996; Zachara et al., 2004; Himmelfarb, 2005; Dominguez et al., 2006; Seiler et al., 2008). This review is to summarize the relationship between renal fibrosis and ferroptosis from the perspective of iron metabolism and lipid peroxidation.

## 2 Mechanism of Ferroptosis

### 2.1 Oxidative Stress and Lipid Peroxidation in Ferroptosis

Ferroptosis is a regulated form of cell death characterized by iron-dependent oxidative stress induced lipid peroxidation (Stockwell et al., 2017). In general, ferroptosis is mainly caused by the inactivation of cellular antioxidant system; it is caspase- and necrosome-independent, which is precisely regulated at epigenetic, transcriptional, post-transcriptional and post-translational levels (Galluzzi et al., 2018).

#### 2.1.1 Oxidative Stress

Oxidative stress is s a disruption of redox signaling and control (Jones, 2006), resulting from either excessive free radicals or insufficient degradation of these free radicals. Oxidative stress is a causative factor in the pathogenesis and pathophysiology of numerous degenerative disorders.

Firstly, ROS and reactive nitrogen species (RNS) are the most common oxidative compounds, which can mediate oxidative stress under various pathophysiological conditions, and are removed by antioxidant defense mechanisms. Secondly, reduced glutathione, is an essential intracellular antioxidant synthesized by the ATP-dependent cytosolic enzymes glutamate-cysteine ligase (GCL) and glutathione synthetase (GSS) in a two-step synthesis of cysteine, glutamate and glycine (Stockwell et al., 2017). Thirdly, extracellular cystine and intracellular cysteine are essential for the maintenance of glutathione biosynthesis and for the inhibition of a type of cell death, which can also be prevented by iron chelators or lipophilic antioxidants (α-tocopherol) (Bannai et al., 1977; Murphy et al., 1989). Cysteine is imported into cells through the oxidized form-cystine of system Xc^−^ where cystine is immediately reduced to cysteine within the cells. Finally, GSH is a major soluble non-enzymatic antioxidant, which relieves the oxidative damage and protects cellular macromolecules, including membrane lipids, against ROS by donating electrons to free radicals. This donation leads to its own oxidation, which converts GSH into glutathione disulfide (GSSG). In this process, glutathione peroxidases (GPXs) play an antioxidant role and converts potentially toxic lipid hydroperoxides (L-OOH) to non-toxic lipid alcohols (L-OH) by buffering H_2_O_2_ with GSH (Battin and Brumaghim, 2009).

#### 2.1.2 Lipid Peroxidation

**FIGURE 1:**
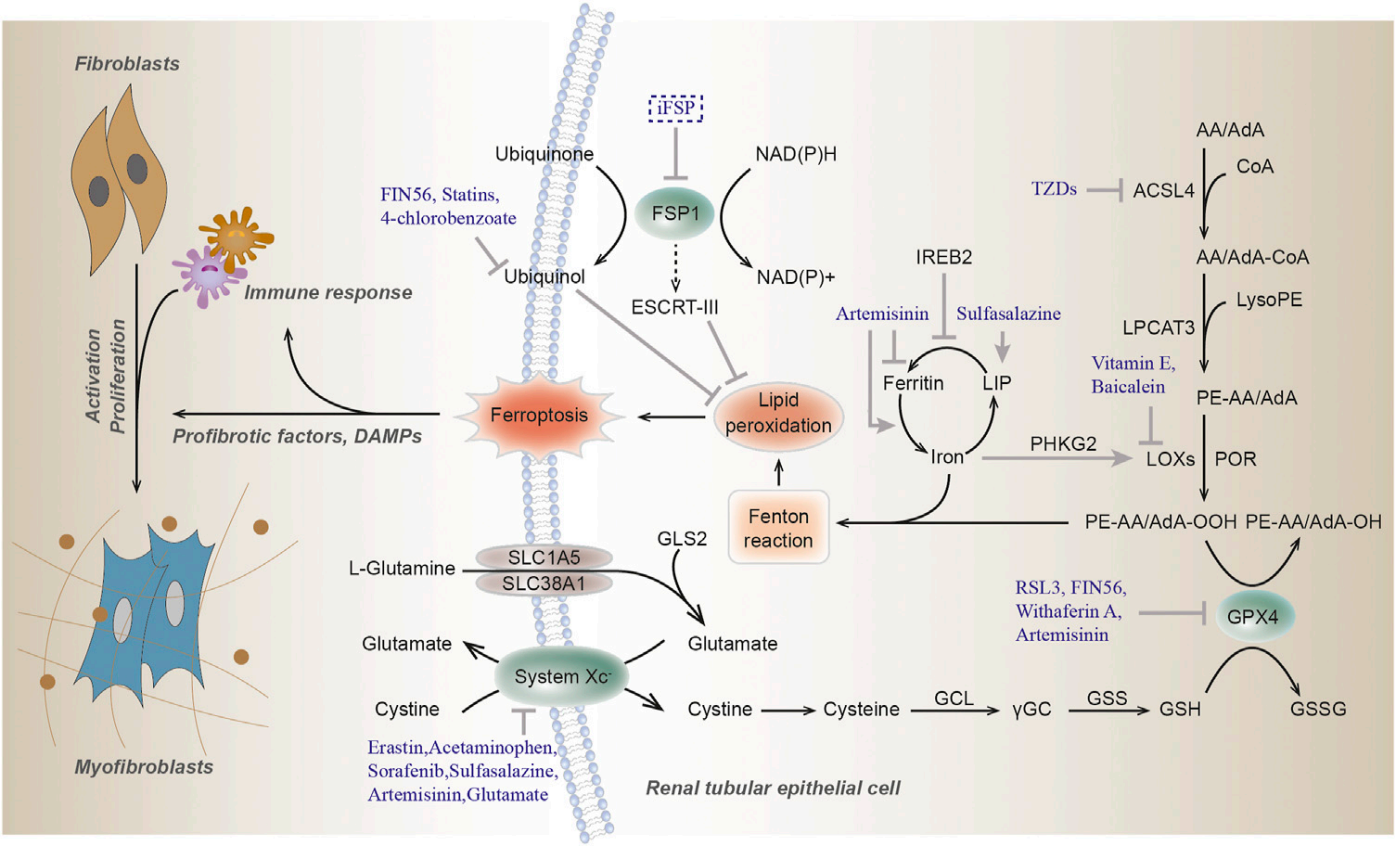
Molecular mechanisms of ferroptosis. Ferroptosis is triggered by lethal lipid peroxidation caused by accumulation of intracellular free iron and/or dysfunction of antioxidant system. The commonly used ferroptosis regulators are shown here. Arrows indicate activation, while blunt lines indicate inhibition. GSH, glutathione; GPX4, glutathione peroxidase 4; GSSG, glutathione disulfide; GCL, glutamate-cysteine ligase; γGC, γ-glutamyl cysteine; GSS, glutathione synthetase; AA, arachidonic acid; AdA, adrenic acid; ACSL4, acyl-CoA synthetase 4; TZDs, thiazolidinediones; LPCAT3, lysophosphatidylcholine acyltransferase 3; LOXs, lipoxygenase; POR, cytochrome P450 oxidoreductase; FSP1, Ferroptosis suppressor protein 1; IREB2, iron responsive element binding protein 2; PHKG2, phosphorylase kinase G2; LIP, labile iron pool; GLS2, glutaminase 2; SLC1A5, solute carrier family 1 member 5; SLC38A1, solute carrier family 38 member A; NAD(P)H, nicotinamide adenine dinucleotide phosphate; ESCRT-III, endosomal sorting complex required for transport-III; RSL3, (1S,3R)-RSL3.

Oxidative stress might impact various macromolecules including lipids, proteins and nucleic acid, leading to the formation of oxidative damage products. Lipid peroxidation, broadly defined as the insertion of hydroperoxyl group into a lipid, has recently been identified as a major driver of ferroptosis (Yang and Stockwell, 2016). This process can be induced by a variety of oxidants (superoxides, H_2_O_2_, and highly reactive hydroxyl radicals) or by exposure to xenobiotics and environmental pollutants. As the main component of cellular membranes, lipids have an indispensable role in maintaining cell structural integrity. Polyunsaturated fatty acids (PUFAs), especially arachidonic acid (AA) or adrenic acid (AdA), are most susceptible to peroxidation, which is quite important for the maintenance of cellular membrane fluidity (Figure 1; de Carvalho and Caramujo, 2018).

In the non-enzymatic pathway, lipid peroxidation can be mediated by carbon- and oxygen-centered radicals initiated by Fenton reaction (see below) or it can also be carried out in an orderly manner by enzyme pathway. Acyl-CoA Synthetase Long Chain Family Member 4 (ACSL4) catalyzes the addition of CoA to the long-chain polyunsaturated bonds of AA or AdA to produce AA or AdA acyl Co-A derivatives, thereby promoting the esterification of PUFA into phospholipids (Kagan et al., 2017). It incorporates cellular membranes with PUFAs and drives ferroptosis through the accumulation of oxidized cellular membrane phospholipids (Doll et al., 2017). Then these derivatives are esterified into phosphatidylethanolamines (PEs) containing AA or AdA (AA-PE or AdA-PE) by Lysophosphatidylcholine Acyltransferase 3 (LPCAT3). Lipoxygenases (LOXs) form a family of iron-containing lipid peroxide enzymes with highly conserved Fe-coordination sphere positioned in a helical core and shows the substrate specificity (Newcomer and Brash, 2015). According to the location specificity of arachidonic acid oxidation, lipoxygenase was named and classified as ALOXE3 (arachidonate lipoxygenase 3), ALOX5 (arachidonate 5-lipoxygenase), ALOX12 (arachidonate 12-lipoxygenase, 12S type), ALOX12B (arachidonate 12-lipoxygenase, 12R type), ALOX15 (arachidonate 15-lipoxygenase), and ALOX15B (arachidonate 15-lipoxygenase type B) in humans. Finally, AA-PE and AdA-PE, were oxidized by lipoxygenases (LOXs) to generate lipid hydroperoxides, thus proximately execute ferroptosis. The antioxidant α-tocopherol and α-tocotrienol could regulate ferroptosis by suppressing the LOX, then generating doubly and triply-oxygenated (15-hydroperoxy)-diacylated phosphatidylethanolamine (PE) species (Kagan et al., 2017). Furthermore, cytochrome P450 oxidoreductase (POR) were recently reported to generate lipid hydroperoxides by an ALOX-independent manner (Figure 1; Zou et al., 2020).

The GPX4, an antioxidant enzyme and a central blocker of ferroptosis, is the only glutathione peroxidase that uses GSH as a co-substrate to reduce phospholipid hydroperoxides to hydroxy phospholipid in the membranes (Yang et al., 2014). Decreased detoxification of GPX4, depletion of GSH and increased LOXs activity are the main driving factors of ferroptosis (Seiler et al., 2008; Yang and Stockwell, 2016). Selenium is a component of selenocysteine at the catalytic site of GPX4. Similar to ferrostatin-1, selenium has a strong inhibitory effect on cerebral hemorrhage and can reduce the concentration of ferroptosis, which is a specific ferroptosis inhibitor (Ingold et al., 2018). In addition, lipophilic antioxidant ubiquinone (CoQ10) exerts a fundamental role in the mitochondrial electron transport chain and can hamper ferroptosis in a GSH-independent manner (Doll et al., 2019). Glutamate is converted from glutamine by glutaminase and is required for the induction of ferroptosis during serum-induced injury after amino acid starvation (Gao et al., 2015; Altman et al., 2016). Then glutamate can also be exchanged for cystine via xCT. Ferroptosis suppressor protein 1 (FSP1), also named as apoptosis-inducing factor mitochondria associated 2 (AIFM2), acts as an NADH-dependent oxidoreductase to catalyze oxidase ubiquinone into reduced ubiquinol and prevents lipid peroxides and incorporation into membranes and lipoproteins, in parallel to the canonical glutathione-based GPX4 pathway (Bersuker et al., 2019; Doll et al., 2019). The newly found Dihydroorotate dehydrogenase (DHODH), inhibits ferroptosis in the mitochondrial intima by reducing the ubiquitin ketone to ubiquitin alcohol, operating in parallel to mitochondrial GPX4, but not to cytosolic GPX4 or FSP1 (Mao et al., 2021; Figure 1).

### 2.2 Iron Homeostasis and Ferroptosis

Iron is an important trace element that plays an important role in many key biological processes. Iron is a key cofactor in electron transfer processes and redox reactions because of its ability to switch between specific oxidative forms; however, iron’s redox potential might lead to cytotoxicity when iron is overloaded (Gozzelino and Arosio, 2016).

#### 2.2.1 Iron Overload Induces Ferroptosis

The excessive intracellular labile irons can directly participate in the redox cycle, providing electrons to expedite ROS production and lipid peroxidation via the Fenton reaction, a series of reactions in which this labile iron reacts with endogenous hydrogen peroxide or superoxide to form oxygen centered radicals (Fe2+ + H2O2 → Fe3+ + OH- + ⋅ OH) (Koppenol, 1993; Neyens and Baeyens, 2003). GSH is necessary for the assembly of Fe-S clusters and can act as a coordinating ligand for [2Fe-2S] clusters when bound to a glutaredoxin scaffold (Hider and Kong, 2013). Interestingly, studies in yeast support the prime actor of GSH in maintaining iron balance during iron-sulfur cluster assembly, challenging the traditional view of its primary role in redox balance (Kumar et al., 2011).

**FIGURE 2:**
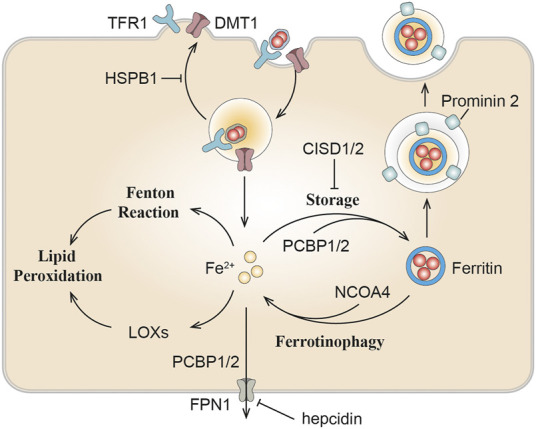
The role of iron metabolism in ferroptosis. Iron homeostasis or transportation including can influence ferroptosis sensitivity. DMT1, divalent metal transporter 1; TFR1, transferrin receptor 1; NCOA4, nuclear receptor coactivator 4; PCBP, Poly(rC) binding protein; LOXs, lipoxygenase; HSPB1, heat shock protein beta-1; CISD, CDGSH iron-sulfur domain.

Compelling evidence indicated that ferroptosis can be motivated by elevated iron levels and inhibited by iron chelation (Dixon et al., 2012). Several proteins impacting iron homeostasis or transportation can influence the sensitivity of ferroptosis (Figure 2; Dixon et al., 2012; Sun et al., 2015; Yuan et al., 2016; Kim et al., 2018). Ferritin plays a major role in protecting against ferroptosis through storing intracellular iron (Fang et al., 2020). It further emphasizes the essentiality of iron in ferroptosis, which requires transferrin and TFR1 as specific markers of ferroptosis (Gao et al., 2015; Feng et al., 2020). DMT1 inhibition could block the translocation of lysosomal iron, leading to the accumulation of lysosomal iron and cell death with ferroptotic features (Song et al., 2021). NCOA4-mediated ferritinophagy promotes ferroptosis by degradation of ferritin (Hou et al., 2016). In kidney, the liver-derived hormone hepcidin serves as a promising therapeutic target in conditions such as post-cardiopulmonary bypass AKI and delayed graft function after renal transplantation (Scindia et al., 2019). Reportedly, it could promote erastin-induced ferroptosis by degrading ferroportin, the sole iron export protein (Geng et al., 2018). Recently, prominin 2 has been found to facilitate the formation of ferritin-containing multivesicular bodies (MVB) and exosomes in mammary epithelial, thus driving ferroptosis resistance (Belavgeni et al., 2019; Brown et al., 2019). Heat shock protein beta-1 (HSPB1) can inhibit TfR1 recycling (Chen et al., 2006), its up-regulation can confine erastin-induced iron uptake through suppressing the ferroptosis (Sun et al., 2015). In addition to participating in Fenton reaction, iron is also transported to several iron-containing enzymes involved in lipid peroxidation, thus in turn actuating ferroptosis. For instance, phosphorylase kinase G2 (PHKG2) plays a significant role in promoting iron accumulation and regulating iron availability to LOXs (Yang et al., 2016). CDGSH iron-sulfur domain (CISD) has also been shown to bind a redox-active [2Fe-2S] cluster and participates in regulating iron storage and redox reactions (Fidai et al., 2016). Down-regulating CISD1 level can promote iron-mediated intramitochondrial lipid peroxidation contributing to erastin-induced ferroptosis (Yuan et al., 2016). Silencing CISD2 gene can raise lipid ROS production and mitochondrial iron content, making drug-resistant head and neck cancer cells sensitive to sulfasalazine-induced ferroptosis (Kim et al., 2018). Accumulation of iron responsive element binding protein 2 (IREB2) can induce iron aggregation, thus contributing to the ferroptosis (Li Y. et al., 2020), while the silencing of IREB2 gene leads to mutual changes in the expression of known iron absorption, metabolism and storage genes TFR1, iron-sulfur cluster assembly enzyme (ISCU), FTH1, FTL and in erastin sensitivity (Salahudeen et al., 2009; Vashisht et al., 2009).

## 3 Ferroptosis in Chronic Renal Injury and Fibrosis

### 3.1 Lipid Peroxidation in Chronic Kidney Disease

The final common pathological denominator in the case of chronic kidney injury is renal fibrosis, and the extent of tubulointerstitial fibrosis is the best predictor of renal survival for CKD patients (Zeisberg and Neilson, 2010). Oxidative stress is present from the early stages of CKD and continues to increase as kidney function deteriorate due to antioxidant consumption and increased ROS production (Sureshbabu et al., 2015; Daenen et al., 2019; Duni et al., 2019; Gyuraszova et al., 2019).

Diverse renal abnormalities can trigger oxidative stress and accelerate the kidney disorders. Fibrotic and damaged tubular and glomerular cells in CKD may enhance ROS production, which is a major agent of oxidative stress (Daenen et al., 2019). Compared with healthy control group, the levels of GPX and GSH in renal cortex of CKD model rats and CKD patients were significantly decreased, while the levels of ROS and MDA were significantly increased (Zachara et al., 2004; Tbahriti et al., 2013). Lipid aldehydes, the end product of many oxidation reactions, including 4-hydroxy-2-hexenal (4-HHE) and 4-hydroxy-2-nonenal (4-HNE) (Himmelfarb, 2005), and their plasma concentrations are significantly higher in CKD patients (Soulage et al., 2020). Malondialdehyde (MDA), is the final product of peroxidation of polyfluorinated fatty acids and an indicator of ferroptosis (Kim et al., 2018), which is commonly raised in the serum of CKD patients (Taccone-Gallucci et al., 1989; Pei et al., 2016). These markers, including 4-HHE, 4-HNE, and MDA have all been widely used in the detection of ferroptosis recently. In HEK-293T cells, GPX4 was shown to block TNFα-mediated activation of NF-кB (Li et al., 2018). Moreover, recent researches have reflected that ferroptosis could facilitate renal damage in diabetic nephropathy (DN), and lipid peroxidation is increased in DN mice, and the expressions of ACSL4, SLC7A11, and SLC3A2 are up-regulated, while GPX4 is down-regulated (Wang Y. et al., 2020). ACSL4 inhibitor rosiglitazone alleviated renal impairment in DN by suppressing ferroptosis (Wang Y. et al., 2020). It is established that pathological changes of DN include renal hypertrophy, enlargement of glomerular capillaries, mesangial expansion, and glomerular basement membrane thickening caused by ECM deposition, which ultimately lead to inflammation, endothelial dysfunction, and renal fibrosis (Jha et al., 2016).

### 3.2 Iron Metabolism in Kidney

#### 3.2.1 Iron Homeostasis in Kidney

**FIGURE 3:**
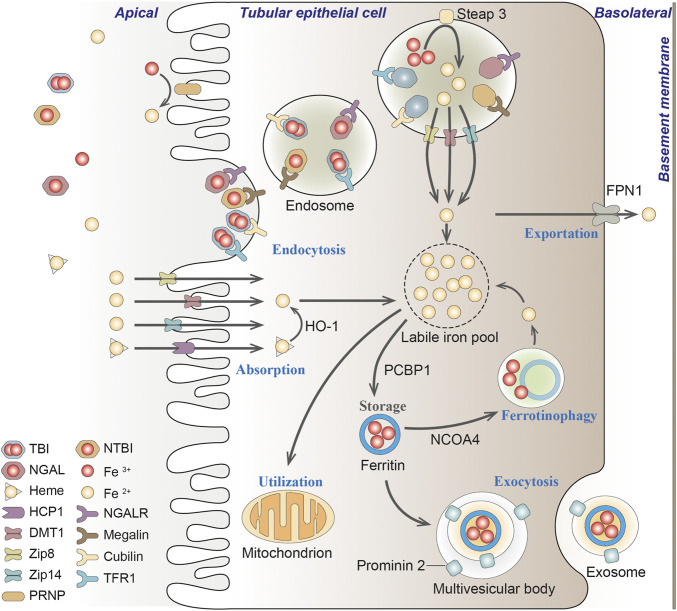
Iron transportation in renal tubules. Filtered iron is almost completely reabsorbed in renal tubular, where it would be then stored in ferritin, utilized in the mitochondria or exported from the cell. TBI, transferrin-bound iron; NTBI, non-transferrin-bound iron; NGAL, neutrophil gelatinase-associated lipocalin; HO-1, heme oxygenase-1; PCBP1, Poly(rC) binding protein 1; NCOA4, nuclear receptor coactivator 4; FPN1, protein ferroportin 1; HCP1, heme carrier protein 1; DMT1, divalent metal transporter 1; Zip8, zinc transporter ZIP8; Zip14, zinc transporter ZIP14; PRNP, prion protein; NGALR, neutrophil gelatinase-associated lipocalin receptor; TFR1, transferrin receptor 1.

Normally, nutritional iron, which consists of both ferric iron (Fe^3+^) and ferrous iron (Fe^2+^), is absorbed by duodenal enterocytes. Fe^3+^ could bind to transferrin (TF) on the cell membrane to form transferrin-bound iron (TBI), which is captured by transferrin receptor 1 (TFR1) into the endosomes. Intracellular Fe^3+^ is reduced to Fe^2+^ by iron reductase (represented by Steap3). Fe^2+^ would then be released into the labile iron pool (LIP) in the cytoplasm by the divalent metal transporter 1 (DMT1) (Dhungana et al., 2004; Knutson, 2007). Endosomal Fe^3+^ is quickly reduced to Fe^2+^ by an oxidoreductase activity. Excess iron ions are either released through the basal membrane protein ferroportin 1 (FPN1) to the circulation or stored in ferritin, the major iron storage protein (Arosio et al., 2009). Ferritin is a spherical heteropolymer capable of storing 4,500 iron atoms, consisting of a ferritin heavy chain (FTH1) and a ferritin light chain (FTL), both of which can sequestrate and store iron in a non-toxic and bioavailable form. Iron is incorporated into ferritin via ferritin iron pores as Fe^2+^, which could be formed by intestinal absorption or erythrocyte degradation. FTH1 catalyzes the oxidation of Fe^2+^ to Fe^3+^, while FTL promotes the nucleation of ferrihydrite, enabling inert deposits of Fe^3+^, which can hardly be used in cells or generate ROS (Philpott, 2018). Poly(rC) binding proteins (PCBPs) (especially PCBP1 and PCBP2), are important iron chaperones to regulate the metalation of iron-containing proteins and the storage and export of Fe^2+^ (Shi et al., 2008). They directly bind ferritin and deliver labile irons to ferritin for storage (Philpott et al., 2017). For intracellular utilization, iron needs to be released from ferritin and reduced to Fe^2+^ again. Ferritin degradation mediated by nuclear receptor coactivator 4 (NCOA4) is required for the maintenance of mitochondrial respiratory activity, respiratory chain complex assembly, mitochondrial iron storage, and membrane potential (Figure 3; Fujimaki et al., 2019). However, it has been reported that iron excess in red blood cells in turn triggers the ubiquitin-mediated NCOA4 degradation by the E3-9 ligase HERC2 (Ryu et al., 2018).

In the kidney, circulating iron can be filtered through the glomeruli into the tubules under physiological conditions (Zhang et al., 2007; Smith and Thevenod, 2009). The filtered iron is almost completely reabsorbed in the renal tubules, and then stored in ferritin, utilized in the mitochondria or exported from the cell (Smith and Thevenod, 2009). If systemic iron is overloaded and transferrin is mostly saturated, iron would loosely bind to other filterable non-transferrin proteins, such as neutrophil gelatinase-associated lipocalin (NGAL), albumin, hemoglobin, myoglobin and hepcidin, or small molecules such as citrate, acetate, and phosphate (Swinkels et al., 2006; Smith and Thevenod, 2009; Brittenham, 2011; Kroot et al., 2011; Paragas et al., 2012; de Swart et al., 2016; Rosa et al., 2017; Figure 3).

The reabsorption of filtered transferrin-bound iron (TBI), hemoglobin and myoglobin in the renal tubules is mainly through megalin-dependent cubilin-mediated endocytosis via binding to TFR1 expressed in the apical membrane (Kozyraki et al., 2001). Renal TFR1 expression is decreased during parenterally induced iron loading (Weiss et al., 2018), but increased under low iron conditions (Smith et al., 2019). NGAL is a promising biomarker for acute or chronic kidney disease, where iron binds to NGAL and is enucleated by neutrophil gelatinase-associated lipid calin receptor (NGALR) in distal tubules (Langelueddecke et al., 2012). Heme is reabsorbed through the heme importer, namely, heme carrier protein 1 (HCP1), in proximal and distal tubules (Le Blanc et al., 2012). Transporters in different nephron segments that promote the uptake of other non-transferrin proteins binding iron on the plasma membrane has not been fully elucidated (Thévenod and Wolff, 2016). Reportedly, zinc transporter ZIP8 (Zip8), zinc transporter ZIP14 (Zip14) and DMT1 were present in the apical membrane of human proximal and distal tubules (Canonne-Hergaux and Gros, 2002; Veuthey et al., 2014; van Raaij et al., 2018b). The endocytic iron is released from the transport protein, and is then reduced and exported to the cytosol mainly through DMT1, where Fe^3+^ is excluded under the excitation of an H^+^ electrochemical potential gradient, and potentially pass through the Zip14 or Zip8 (He et al., 2006; Pinilla-Tenas et al., 2011). Since DMT1 optimally transports ferrous iron at pH 5.0–6.0, whereas Zip8 and Zip14 at pH 6.5–7.5 (He et al., 2006; Pinilla-Tenas et al., 2011), it is speculated that DMT1 may mainly facilitate the uptake of free iron distal tubules, while Zip8 and Zip14 may play a better role in the proximal tubules (Figure 3; van Swelm et al., 2020).

There is no evidence for basolateral uptake of TBI, heme, hemoglobin, or non-transferrin-bound iron (NTBI) in the basolateral renal tubules so far, whereas the iron exporter-ferroportin exists in the basolateral membrane of proximal tubules (van Raaij et al., 2018a). As a master regulator of iron homeostasis, hepcidin regulates this ferroportin-mediated iron export and intracellular H-ferritin levels (Vyoral and Jiri, 2017). In podocytes, hemoglobin can be taken up by megalin-cubilin endocytosis catabolized by heme oxygenase-1 (HO-1), thus resulting in increased oxidative stress (Rubio-Navarro et al., 2018; Duann et al., 2019). In addition, iron transport and regulation also exist in mesangial cells, since ferritin, TFR1, HO-1, and IRP1 are all expressed in these cells (Cheng et al., 2015; Liu and Templeton, 2015).

#### 3.2.2 Iron Overload and Toxicity in Kidney

**FIGURE 4:**
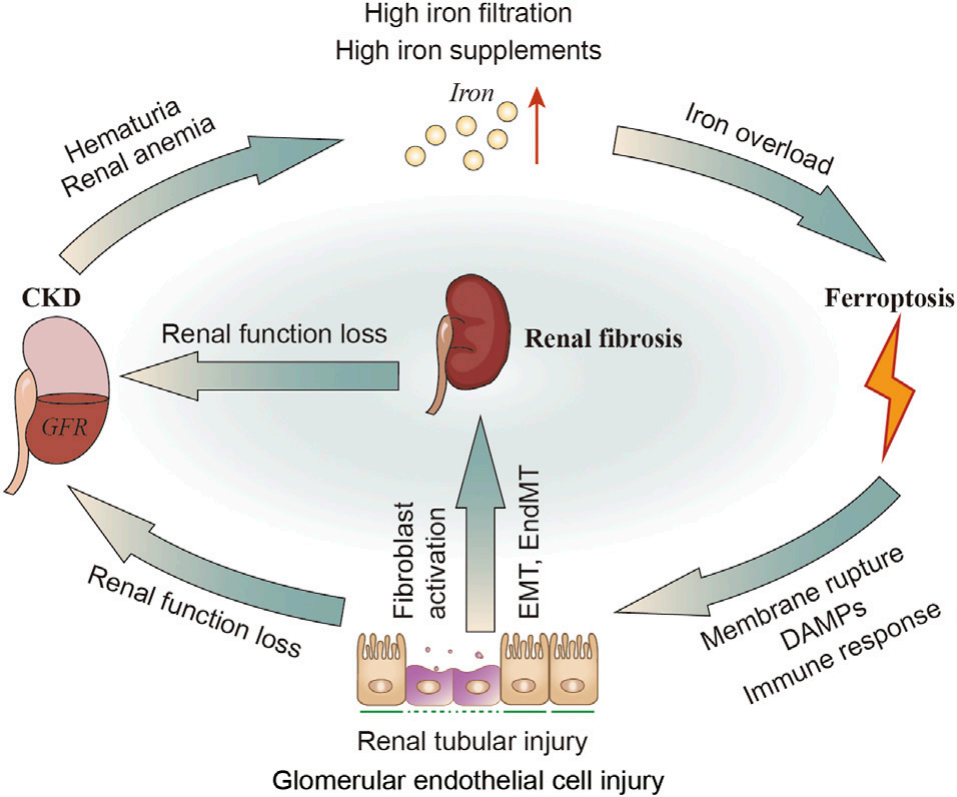
The relationship of CKD, iron metabolism, ferroptosis in renal tubular cells and renal fibrosis. In patients with CKD, hematuria and renal anemia could cause increased iron filtration in renal tubules and excessive exogenous intake of iron. Iron overload may induce ferroptosis, leading to renal injury and DAMPs signals release as well as inflammatory and immune responses. The injured tubular can then activate fibroblasts or directly transform into myofibroblasts through EMT. Kidney injury or renal fibrosis can further reduce the GFR. DAMPs, damage-related molecular patterns; GFR; glomerular filtration rate.

Iron plays a key role in mitochondria, promoting oxidative stress and ROS production, leading to cell apoptosis, mitochondrial damage and vascular calcification (Nakanishi et al., 2020). In 1988, the deleterious role of iron in renal disease was first described as being involved in free radical mediated renal damage after ischemia and lipid peroxidation, while iron chelators can reduce these injury (Paller and Hedlund, 1988), suggesting the exsistance of ferroptosis. Mitochondria are extremely dependent on iron metabolism and are the target of iron transport in these cells. The tubular epithelium possesses abundance of mitochondria to support active transport, but quite susceptible to hypoxia, toxic compounds, proteinuria, metabolic disorders. Kidneys can be exposed to toxic levels of bound iron (e.g., transferrin), or free iron, due to redundant filtration of iron into renal tubules, alterations in cellular iron localization or compartmentalization in the kidney (Martines et al., 2013; van Raaij et al., 2018b; Figure 4).

It was found that kidney and urinary iron content are elevated in patients with CKD (Sears et al., 1966; Wang et al., 2001; Shah et al., 2007; van Raaij et al., 2018b) and animal models of CKD (Nankivell et al., 1994; Ueda et al., 1996; Harris and Tay, 1997; Naito et al., 2015). Altered molecular iron handling may lead to iron deposition in kidney (van Raaij et al., 2018b). When these iron or iron-containing molecules exceed the storage capacity of ferritin or the cell’s ability to export iron, they damage renal tubular cells (Sponsel et al., 1996; Zager and Burkhart, 1997; Martines et al., 2013; Naito et al., 2015). Exposure to either the Fe^2+^ or Fe^3+^ (100 μM, 30–80 h) may impair healing of denuded areas made within confluent monolayers of renal tubular epithelial cell lines (Sponsel et al., 1996). Fe^2+^ (FeSO_4_ 100 μM) can stimulate H_2_O_2_ production in renal tubular cells *in vitro* (Zager and Burkhart, 1997). Fe^2+^ can also cause cytopathic changes including numerous iron-particles-containing lysosomes and distortional mitochondria (Sponsel et al., 1996; Figure 4). Apart from the tubules, cultured human glomerular endothelial cells (HGECs) have been shown to express TFR1, FPN1 and DMT1. The endothelial injury factor-Ang II could induce iron incorporation followed by an increase in labile iron in HGECs (Tajima et al., 2010). Iron chelators have been confirmed to improve renal function in patients with glomerular disease, and minimize the reactivity of iron and remove iron from the kidneys or relieving iron-related oxidative stress (Rajapurkar et al., 2013). Iron restriction can also delay the progress of DN in diabetic rats (Matsumoto et al., 2013). In addition, administration of iron chelation can alleviate renal interstitial fibrosis and suppress the increments of collagen type III and TGF-β (Naito et al., 2015).

Iron-mediated cell injury or ferroptosis is considered as a core of the pathogenesis of acute kidney injury (Scindia et al., 2019). Iron homeostasis is becoming the focus of new therapeutic interventions for kidney diseases. Although iron overload or toxicity are not equivalent to ferroptosis, there is reason to believe that these “iron induced oxidative stress or lipid peroxidation” are at least related to ferroptotic events.

### 3.3 Ferroptosis in Renal Fibrosis

ROS play a wide role in the oxidation of lipids, proteins, and DNA, resulting in damage to the tubules and vascular endothelium, thus secreting profibrotic factors (Pisani et al., 2013; Daenen et al., 2019). Inhibition of oxidative stress has always been an anti-fibrotic target (Wang et al., 2019). Oxidative stress can cause fibroblast/pericyte activation (Bondi et al., 2010), tubular epithelial-mesenchymal transition (EMT) (He et al., 2015; Song et al., 2018), fibrocyte/macrophage recruitment (Wagner et al., 2012; Lee et al., 2016), and endothelial-mesenchymal transition (EndMT) (Echeverria et al., 2013; Montorfano et al., 2014). Reportedly, HK2 (human kidney tubular cell) has been reported to secrete profibrotic factors and facilitate fibroblast proliferation and activation during ferroptosis (Zhang et al., 2021). Oxidative stress induced glomerular endothelial cell dysfunction (Sol et al., 2020), mesangial sclerosis (Lu et al., 2018), podocyte abnormality (Okamura and Pennathur, 2015; Zschiedrich et al., 2017), and parietal epithelial cell injury (Odaci et al., 2015; Su et al., 2015) can lead to glomerulosclerosis (Tang et al., 2011; Su et al., 2019). Increased production of ROS (including OH^−^, H2O2, lipid peroxides, and superoxide anion free radicals) in podocytes can directly damage podocytes themselves, which consequently meditate glomerular injury (Gwinner et al., 1997; Marshall et al., 2006). Numerous pathologic processes mediated by oxidative stress associated factors can stimulate glomerular endothelial cells to undergo EndMT, which is consistent with increased mesenchymal marker expression including α-smooth muscle actin (αSMA) and fibroblast specific protein 1, and the production of ECM proteins (Liang et al., 2016; Dejana et al., 2017; Figure 4).

Although oxidative stress is known as an inducer for ferroptosis, we have to notice it is the “lipid ROS” and subsequently membrane rupture that represent the occurrence of ferroptosis, instead of the “ROS” (Dixon et al., 2012). Ferroptosis triggers inflammatory and immune responses through the release and activation of damage-related molecular patterns (DAMPs) signals, which makes it to be an attractive therapeutic target for renal fibrosis especially renal graft fibrosis (Linkermann et al., 2013; Martin-Sanchez et al., 2017). Synchronous tubule necrosis can be induced in renal ischemia/reperfusion (I/R) injury model, oxalate crystal-induced AKI model and diabetic nephropathy (DN) tubule cell death model (Linkermann et al., 2014; Wang Y. et al., 2020; Li S. et al., 2021). Erastin or TGF-β can induce ferroptosis, promote fibroblast-to-myofibroblast differentiation resulting in fibrosis (Gong et al., 2019). Recent studies have shown that ureteral obstruction can induce ferroptosis in renal tubular epithelial cells *in vivo*, and HK-2 cells (human proximal tubular cell line) to secrete various profibrotic factors which further promoted the proliferation and activation of fibroblast during ferroptosis (Zhang et al., 2021). Here, we first briefly summarize the role of several key regulators of ferroptosis in renal fibrosis, though the specific mechanism of these prooxidative factor in triggering ferroptosis and mediating renal fibrosis has far been elucidated.

#### 3.3.1 HO-1

HO-1, an inducible enzyme, catabolizes the heme group into carbon monoxide (CO) and biliverdin, which can further convert to bilirubin, and ferrous iron. HO-1 induction could protect the kidney from oxidative stress showing anti-inflammatory and anti-apoptotic activity in DN (Abo El Gheit and Emam, 2016), which was also proved to reduce glomerular sclerosis and intimal hyperplasia, being a therapeutic target to improve the long-term outcomes after kidney transplantation (Bedard et al., 2005; Corona et al., 2020). Additionally, compared with wild-type mice, HO-1^−/−^ mice had higher levels of renal fibrosis (Kovtunovych et al., 2010), and more susceptible to myocardial I/R (Abo El Gheit and Emam, 2016). Absence of HO-1 was associated with immune response deficiency, such as Th1-weighted shift in cytokine responses (Kapturczak et al., 2004), and defects in erythrophagocytosing macrophages (Kovtunovych et al., 2010), thus supporting it a protective role in kidney diseases. However, activation of HO-1 triggers ferroptosis through iron overloading and excessive lipid peroxides, thus promoting renal fibrosis area and collagen I in DN mice (Chang et al., 2018; Feng et al., 2021). Increased HIF-1α/HO-1 level, iron content, ferritin level and lipid peroxides were detected in this DN kidneys, indicating that ferroptosis might enhance DN and damage renal tubules through HIF-1α/HO-1 pathway (Feng et al., 2021). Researches on neurodegenerative diseases and neoplastic disorders also demonstrated HO-1 has pro-ferroptotic effects on cells through iron accumulation and pro-oxidant effects (Chang et al., 2018; Li R. et al., 2020; Fernández-Mendívil et al., 2021).

Resultingly, though induction of HO-1 has been a potential therapeutic target in kidney diseases, attention must be paid to the safe range of HO-1 induction, owing to the pro-ferroptotic role of HO-1 in raising intracellular ferrous load and redox homeostasis (Chang et al., 2018; Li R. et al., 2020; Fernández-Mendívil et al., 2021).

#### 3.3.2 NOX4

NADPH oxidase (NOXs) plays the most important role in many enzyme systems involved in ROS production in the kidney (Jha et al., 2016). Nox4 is a major source of ROS in the renal cells, linking with myofibroblast differentiation and tubulointerstitial fibrosis through various pathways. Although Nox4 is expressed in a variety of kidney cells, including epithelial, endothelial, podocytes, mesangial cells, and fibroblasts, its role in fibrosis is controversial (Yang et al., 2018). Inhibition of NOX4 could protect from ferroptosis in various cells via suppressing H_2_O_2_ and lipid peroxides production (Dixon et al., 2012; Wang et al., 2018; Chen et al., 2019). Manipulating Nox4 expression in mice proximal tubules cells could upregulate renal fibronectin and TGF-β level (Sedeek et al., 2010). It is confirmed that rapamycin can decrease MDA and NOX4 levels, inhibit the NADPH oxidase activity, and elevate the GSH level and SOD activity, thus inhibiting the ROS production of mesangial cells in DN (Lu et al., 2018). Nrf2 is known to regulate the antioxidant response and the activity of several ferroptosis-related proteins, involving iron homeostasis, intermediate metabolism, and GSH metabolism; Nrf2-mediated up-regulation of HO-1, as described above, could facilitate heme degradation, free iron release, and lipid peroxidation, thus inducing ferroptosis (Dodson et al., 2019; Fang et al., 2019). The up-regulation of Nrf2 expression by Fenofibrate can inhibit iron droop, thus alleviating renal pathological changes in DN mice (Li S. et al., 2021). HIF-1α could negatively regulate ferroptosis induction in human cancer cells (Yang M. et al., 2019), which is also mechanically antithetical to oxidative stress mentioned above (Nlandu Khodo et al., 2012). This is contrary to the traditional view that NOXs promotes ferroptosis by oxidative stress-induced lipid peroxidation (Xie et al., 2016a; Wang J. et al., 2020; Park et al., 2021). Different subcellular localization, cell types, molecular concentrations, disease types or/and stages may be responsible for this functional variability, and more research is warranted on cell-specific and inducible overexpression or knockout systems *in vivo*.

#### 3.3.3 TP53

TP53 (p53) is considered as a tumor suppressor due to its ability to promote cell death or permanently inhibit cell proliferation. P53 can negative regulate SLC7A11, leading to reduced glutathione production and increased ROS (Jiang et al., 2015), target SAT1 which increase the level of ALOX15 (Ou et al., 2016), suggesting that p53 represents a novel inducer of ferroptosis. However, p53 can also regulate GSL2 (glutaminase 2) to influence glutamine metabolism and block dipeptidyl-peptidase-4 (DPP4) activity, which finally defense lipid peroxidation and ferroptosis (Hu et al., 2010; Xie et al., 2017). Reportedly, CRISPR/Cas9 mediated p53 deletion cells were sensitive to ferroptosis, implying that p53 hold a pro-survival function in ferroptosis, which requires p53 targeting CDKN1A (P21) expression (Tarangelo et al., 2018). These context-dependent roles of p53 in ferroptosis may result from fine-tuning its phosphorylation, ubiquitination, methylation, acetylation, and other modifications.

P53 also plays a critical role in non-neoplastic diseases including renal fibrosis and CKD, which acts as a critical co-factor for several key fibrotic and cell cycle effectors including TGF-β1, CTGF, PAI-1, P21 (Kortlever et al., 2006; Yang et al., 2010; Samarakoon et al., 2012). P53 inhibitor pifithrin-α was proved to alleviate renal experimental fibrogenesis consistent with decreased CTGF and TGF-β1 levels (Yang et al., 2010). Interestingly, the p53-dependent P21, shows both effect in ferroptosis and renal fibrosis (Tarangelo et al., 2018; Das et al., 2020), but whether a direct link exists between ferroptosis, renal fibrosis, and P21 has not been reported.

## 4 Ferroptosis Regulators and Renal Fibrosis

### 4.1 Ferroptosis Inhibitors

Many drugs or compounds have been reported to inhibit renal fibrosis *in vitro* (Table 1) or *in vivo* (Table 2) and have also been proved to inhibit ferroptosis (Table 3).

**TABLE 1:** Evidence of ferroptosis inhibitors alleviates renal fibrosis *in vitro*.

Agents	Work concentration	Cell line model	Effects	References
Rosiglitazone	1 μM	NRK-52E (Rat renal tubule epithelial cells)/HK2 Cells (Human Kidney 2)	Blocks ferroptosis by Inhibiting ACSL4-mediated lipid peroxidation	Wang Y et al. (2020)
	1–50 µM	Rat mesangial cells	Causes an anti-fibrotic effect in renal mesangial cells by increasing sphingosine kinase 1 levels	Koch et al. (2012)
Troglitazone	1–20 µM	Rat mesangial cells	Causes an anti-fibrotic effect in renal mesangial cells by increasing sphingosine kinase 1 levels	Koch et al. (2012)
Pioglitazone	10 µM	High glucose-induced profibrotic HK-2 Cell model	Reverses the high glucose-induced profibrotic by intervention of AP-1, TGF-β1, and the extracellular matrix protein fibronectin	Panchapakesan et al. (2005)
	5 µM	TGF-β1-induced HK-2 Cell model	Improves mitochondrial functions and protects against renal fibrosis	Sun et al. (2017)
Vitamin E	100 μM	TGF-β1-induced HK-2 Cell model	Inhibits TGF-β1-induced renal fibrosis	Wang et al. (2010)
Baicalein	20–80 µM	TGF-β1-induced NRK-49F cells	Suppresses the TGF-β1 signaling and inducing mitochondrion-associated myofibroblast apoptosis	Wang et al. (2016); Hu et al. (2017)
Ferrostatin-1	10 μM	TGF-β1-induced HK-2 Cell model	Inhibits TGF-β1-induced expression of α-SMA, col1a1 and fibronectin	Li et al. (2022)
Diphenylene iodonium	20 μM	RMC (Rat kidney mesangial cells)	Suppresses methylglyoxal-induced renal fibrosis by inhibiting superoxide, and expression of TGF-β1 and fibronectin	Ho et al. (2007)
N-acetylcysteine	10 mM	Cisplatin-induced NRK-52E	Decreases cisplatin-induced renal interstitial fibrosis via sirtuin1 activation and p53 deacetylation	Li C et al. (2019)
Puerarin	50 μM or 100 μM	HK2	Ameliorates renal fibrosis by reducing oxidative stress induced-epithelial cell apoptosis via MAPK signal pathways	Zhou et al. (2017)
Trolox	1 μM	Mouse renal CD cell line (M−1)	Blunts prorenin-dependent ROS formation and augmentation of profibrotic factors	Gonzalez et al. (2017)
Triiodothyronine	1 nM	HK2	Inhibits TGFβ1 induced renal tubular epithelial to mesenchymal transition by increasing miR34a expression	Lu et al. (2013)
Linagliptin	100–251 µM	Mouse proximal tubular cell	Attenuates FFA-bound albumin-induced tubular inflammation, fibrosis and apoptosis	Tanaka et al. (2016)
U0126	10 μM	HRPTECs (human renal proximal tubular epithelial cells)	Reverses Interleukin-17A induces renal fibrosis	Weng et al. (2020)
	20 mM	Primary PTECs from mice kidneys and pig kidney proximal tubule (LLC-PK1) cell line	Inhibits TGF-β1-induced renal fibrosis	Chen et al. (2014)
Chloroquine	50 μM	HKC-8 cells (human tubular epithelial cell line)	Reduces renal fibrosis via increasing extracellular urokinase and collagen degradation	Fox et al. (2016)
Liproxstatin-1	0.5 μM	HK2 and fibroblasts	Inhibits the proliferation and differentiation of fibroblasts by inhibiting the release of profibrotic factors released by ferroptotic tubular epithelial cells	Zhang et al. (2021)

**TABLE 2:** Evidence of ferroptosis inhibitors alleviates renal fibrosis *in vivo*.

Agents	Dosage regimen	Animal or patient model	Effects	References
Rosiglitazone	5 mg/kg/day, for 12 weeks; 3 or 30 mg/kg/day for 8 weeks)	Chronic renal allograft dysfunction rats	Inhibits TGF-β signaling and the renal tubular EMT	Kiss et al. (2010); Deng et al. (2019)
	3 mg/kg/day, for 4 weeks	Cyclosporine-induced renal injury rats	Inhibits TGF-β signaling and apoptosis	Chung et al. (2005)
	5 mg/kg/day in drinking water, for 24 weeks	Diabetic nephropathy (DN) rats	Alleviates the glomerulosclerosis and tubulointerstitial fibrosis	Bilan et al. (2011)
	5 mg/kg/day, for 4 weeks	Aristolochic acid nephropathy mice	Reduces renal fibrosis	Shui et al. (2017)
Troglitazone	150 or 300 mg/kg/day, for 14 days	UUO mice	Attenuates renal interstitial fibrosis and inflammation through reduction of TGF-β	Kawai et al. (2009)
	6 mg/g chow for the first 12 weeks and 12 mg/g chow between weeks 13 and 25	Type 2 diabetes mellitus rats	Ameliorates mesangial expansion thus preventing development of glomerulosclerosis	McCarthy et al. (2000)
Pioglitazone	20 mg/kg/day, for 5 weeks	TGF-β overexpressing transgenic mice	Prevents TGF-β induced renal fibrosis by repressing EGR-1 and STAT3	Németh et al. (2019)
	10 mg/kg/day, for 2 weeks	UUO mice	Reduces renal tubulointerstitial fibrosis via miR-21-5p modulation	Han et al. (2010); Sun et al. (2019)
	10 mg/kg/day, for 8 weeks	5/6 nephrectomized rats	Reduces renal fibrosis by downregulating TGF-β1, fibronectin and collagen I	Sun et al. (2017)
	5 mg/kg/day, for 24 weeks	Spontaneously hypercholesterolemic rats	Exerts renoprotective effects and inhibits plasminogen activator inhibitor-1 level	Omasu et al. (2007)
Vitamin E	350 mg/kg, for 2 weeks	UUO mice	Inhibits the TGF-β1/Smad2/3 during EMT	Farias et al. (2020)
	100 mg/kg/day, for 9 weeks	Renal interstitial fibrosis rats	Inhibits renal interstitial fibrosis via alleviation of autophagic stress	Zhao et al. (2018)
	25 mg/kg	Cyclosporine nephrotoxicity rats	Inhibits renal interstitial fibrosis by suppressing renal mRNA expression of COX II, HO I, TGF-β, and osteopontin	Jenkins et al. (2001)
Baicalein	50 or 100 mg/kg/day, for 7 days	UUO mice	Attenuates renal fibrosis by inhibiting inflammation via down-regulating NF-kappaB and MAPK signaling mitochondrion-associated myofibroblast apoptosis	Wang W et al. (2015)
CoQ10 (also known as CoQ6)	20 and 30 mg/kg/day in 2–3 divided doses	Patients with ADCK4-related glomerulopathy	Appeared renoprotective effects with preserved eGFR and reduced proteinuria	Atmaca et al. (2017)
N-acetylcysteine	500 mg/kg/day, for 9–11 weeks	Cisplatin-induced mice	Decrease cisplatin-induced renal interstitial fibrosis via sirtuin1 activation and p53 deacetylation	Li C et al. (2019)
	40 mg/kg/day; 8 weeks	Dilated Cardiomyopathy mice	Attenuates tubulointerstitial and glomerular fibrosis and renal oxidized glutathione levels	Giam et al. (2017)
	250 mg/kg/day, for 7 days	UUO mice	Alleviate angiotensin II-mediated renal fibrosis	Shen et al. (2016)
	150 mg/kg/day, for 4 weeks	Chronic cyclosporine nephropathy mice	Attenuated tubulointerstitial fibrosis in p-AKT/p-FoxO1 pathway	Piao et al. (2013)
Puerarin	50 mg/kg or 100 mg/kg, for 7 days	UUO mice	Ameliorates renal fibrosis by reducing oxidative stress induced-epithelial cell apoptosis via MAPK signal pathways	Zhou et al. (2017)
Zileuton	30 mg/kg/day, for 7 or 14 days	UUO mice	Reduced renal fibrosis through manipulation of 5-lipoxygenase pathway	Montford et al. (2019)
	10 mg/kg, given twice; 1 h before and 12 h after cisplatin	Cisplatin-induced mice	Alleviates cisplatin nephrotoxicity, including tubular necrosis and interstitial fibrosis via renal oxidative/inflammatory/caspase-3 axis	Helmy et al. (2018)
Deferoxamine	100 mg/kg/day, for 7 days	UUO mice	Prevents renal tubulointerstitial fibrosis by regulating TGF-β-Smad signaling, oxidative stress, and inflammatory responses	Ikeda et al. (2014)
Deferiprone	50 mg/kg/day; for 6 months or 150 mg/kg/day; for 9 months	Patients with diabetes and non-diabetic glomerular disease	Exhibits renoprotective role in human	Rajapurkar et al. (2013)
Ferrostatin-1	5 mg/kg, single use	FA-induced mice	Exhibits renoprotective effects	Li Y et al. (2020)
Triiodothyronine	100 μg/kg/day, for 2 weeks	5/6 nephrectomized rats	Attenuates renal fibrosis with reduced NF-кB level and increased HO-1 level	El Agaty, (2018)
FG-4592	10 mg/kg/day, for 2 days	FA-induced mice	Plays a protective role at the early stage of FA-induced renal fibrosis through alleviating ferroptosis via Akt/GSK-3β-mediated Nrf2 activation	Li Y et al. (2020)
Alogliptin	40 mg/kg/day for 10 days	UUO mice	Exhibits renoprotective effects by modulating macrophages M1/2	Uchida et al. (2017)
Linagliptin	3 mg/kg/day, for 16 weeks	5/6 nephrectomized rats	Decreased interstitial fibrosis with decreased urine albumin-to-creatinine ratio	Tsuprykov et al. (2016)
	5 mg/kg/day, for 4 weeks	DN mice	Attenuated renal fibrosis by inhibiting EndMT, apoptosis and blocking the blocked the DPP4/CD32b/NF-кB signaling circuit	Kanasaki et al. (2014); Oraby et al. (2019); Tang et al. (2021)
Vildagliptin	3 mg/kg/day, for 4 weeks	High-fat diet-induced obese rats	Attenuate renal lipid accumulation-induced lipotoxicity and reduced the TGF-β level	Pengrattanachot et al. (2020)
	3 mg/kg/day, for 15 weeks	Zucker Diabetic Fatty (ZDF) rat	Effectively prevent glomerulosclerosis and restored myogenic constriction	Vavrinec et al. (2014)
Carvedilol	Chow supplemented with 2,400 p.p.m., for 6 weeks	Spontaneously hypertensive stroke-prone (SHR-SP) rats	Shows renoprotective effects by inhibiting TGF-β	Wong et al. (2001)
	60 mg/kg/day	Hypertensive rats	Slows down the development of interstitial fibrosis and tubular atrophy	Jovanovic et al. (2005)
Chloroquine	20 mg/kg, 3 times a week, for 15 days	UUO mice and chronic ischemia reperfusion injury mice	Reduces renal fibrosis via increasing extracellular urokinase and collagen degradation	Fox et al. (2016)
Liproxstatin-1	10 mg/kg/d for 14 consecutive days	UUO mice	Attenuates the expression of profibrotic factors	Zhang et al. (2021)

**TABLE 3:** Mechanisms of ferroptosis inhibitors restraining ferroptosis.

Agents	Work concentration	Effects	Model	References
Rosiglitazone	1 μM	Inhibits ACSL4-mediated lipid peroxidation	NRK-52E/HK2 Cells	Wang Y et al. (2020)
	10–30 μM	Inhibits ACSL4-mediated lipid peroxidation	Pfa1 cells (Breast cancer cell line)	Doll et al. (2017); Kagan et al. (2017)
	0.4–0.5 mg/kg	Inhibits ACSL4-mediated lipid peroxidation	Ischemia/reperfusion (I/R) injury mice	Li Y et al. (2019); Xu et al. (2020)
Troglitazone	10–30 μM	Inhibits ACSL4-mediated lipid peroxidation	Breast cancer cell lines	Doll et al. (2017)
Pioglitazone	10–30 μM	Inhibits ACSL4-mediated lipid peroxidation	Breast cancer cell lines	Doll et al. (2017)
	10 μM	Suppresses sulfasalazine-induced ferroptosis	HNC/SNU cell lines	Kim et al. (2018)
Vitamin E	100 μM	Inhibits lipid peroxidation by scavenging ROS	HT-1080/BJ cell lines	Yang et al. (2014); Shimada et al. (2016)
Baicalein	10 μM	Inhibits ALOX-mediated lipid peroxidation	G-401 cell line (a human renal carcinoma cell line)	Yang et al. (2016)
CoQ10	10 μM	May involve reprogramming of lipid metabolism in a way that is not conducive to the execution of ferroptosis	HT-1080 cells	Shimada et al. (2016)
Diphenylene iodonium	1 μM	Inhibits NOX-mediated lipid peroxidation	SHSY5Y cells	Hou et al. (2019)
N-acetylcysteine	1 mM	Inhibits toxic arachidonic acid products of nuclear ALOX5	Primary cortical neurons	Karuppagounder et al. (2018)
	40 mg/kg/day, for 7 days	Inhibits toxic arachidonic acid products of nuclear ALOX5	Collagenase-induced intracerebral hemorrhage mice	Karuppagounder et al. (2018)
	1 mM	Inhibits erastin-induced ferroptosis through supplementing GSH	HT-1080 cells	Yang et al. (2014)
Puerarin	20 μM	Suppresses erastin- or isoprenaline-induced ferroptosis	H9c2 cells	Liu et al. (2018)
	100 mg/kg/day or 200 mg/kg/day, for 12 weeks	Suppresses erastin- or isoprenaline-induced ferroptosis	Heart failure rats	Liu et al. (2018)
Trolox	100 μM	Inhibits lipid peroxidation	HT-1080 cells	Dixon et al. (2012)
Zileuton	10–100 μM	Inhibits lipid peroxidation by suppressing ALOX5	HT22 neuronal cells	Liu et al. (2015)
Deferoxamine	100 μM	Inhibits iron-mediated lipid peroxidation	HT-1080 cells	Dixon et al. (2012)
Deferoxamine	100 mg/kg/day, for 7 days	Inhibits lipid peroxidation via ferroptotic-specific mitochondria genes	Spinal cord injury rats	Yao et al. (2019)
Deferiprone	100 μM	Inhibits iron-mediated lipid peroxidation	LUHMES cells (Lund human mesencephalic cells)	Do Van et al. (2016)
Ferrostatin-1	0.1–10 μM	Scavenge initiating alkoxyl radicals produced by ferrous iron from lipid hydroperoxides	HT-1080/HEK293T/LUHMES cells	Dixon et al. (2012); Do Van et al. (2016); Miotto et al. (2020)
Triiodothyronine	10 μM	Inhibits lipid peroxidation	H9c2 cells	Mishima et al. (2020)
FG-4592	10 mg/kg	Alleviates ferroptosis via Akt/GSK-3β-mediated Nrf2 activation	FA-induced mice	Li Y et al. (2020)
Alogliptin	10 μM	inhibits NOX1-mediated lipid peroxidation	TP53^−/−^ or TP53-deficient CRC cells (HCT116 and SW48)	Xie et al. (2017)
Linagliptin	10 μM	inhibits NOX1-mediated lipid peroxidation	TP53^−/−^ or TP53-deficient CRC cells (HCT116 and SW48)	Xie et al. (2017)
Vildagliptin	10 μM	inhibits NOX1-mediated lipid peroxidation	TP53^−/−^ or TP53-deficient CRC cells (HCT116 and SW48)	Xie et al. (2017)
U0126	10 μM	Inhibits lipid peroxidation independent from MEK activity	MEFs (mouse embryonic fibroblasts)	Gao et al. (2015)
Carvedilol	0.1, 1, and 1 μM	Inhibits lipid peroxidation	H9C2 cells	Mishima et al. (2020)
Chloroquine	50 μM	Blocks ferroptosis at earlier time points, but the inhibitory effect will be gradually lost at later time points	MEFs and HT1080 cells	Gao et al. (2016)

#### 4.1.1 Antioxidants

Several antioxidants can suppress ferroptosis through limiting lipid peroxidation and have been verified to alleviate renal fibrosis from different causes *in vivo* and/or *in vitro* through different targets and mechanisms, including Vitamin E, CoQ10, N-acetylcysteine (NAC), Trolox, Diphenylene iodonium, and Zileuton (Jenkins et al., 2001; Ho et al., 2007; Piao et al., 2013; Shen et al., 2016; Atmaca et al., 2017; Giam et al., 2017; Gonzalez et al., 2017; Helmy et al., 2018; Zhao et al., 2018; Li C. et al., 2019; Montford et al., 2019; Farias et al., 2020).

#### 4.1.2 Iron Chelators

Reportedly, deferoxamine prevents renal interstitial fibrosis in the UUO mice (Ikeda et al., 2014). Long-term oral administration of deferiprone have been shown to have renal protective effects in both diabetic and non-diabetic glomerular disease (Rajapurkar et al., 2013). These two iron chelating agents have anti-iron toxicity at 100 μM (Do Van et al., 2016).

#### 4.1.3 CYP450 Substrate

Certain cytochrome P450 substrate drugs have the anti-ferroptotic effect by scavenging activity against lipid peroxyl radicals in various cell lines including NRK49F (rat kidney fibroblast), NRK52E (rat kidney tubular cell), and HK2 (Mishima et al., 2020). These drugs inhibit the ferroptotic pathological changes of different renal cell types and ameliorates renal damage, indicating the therapeutic promise of this repurposed drugs (e.g., Triiodothyronine, and Carvedilol) (Wong et al., 2001; Jovanovic et al., 2005; Lu et al., 2013; Adedoyin et al., 2018; El Agaty, 2018).

#### 4.1.4 Thiazolidinediones

The PPAR-γ agonists thiazolidinediones (TZDs), including rosiglitazone, troglitazone, and pioglitazone, could prevent ferroptosis probably by inhibiting ACSL4, even in PPARγ KO cells (Doll et al., 2017; Kagan et al., 2017; Wang Y. et al., 2020). However, non-thiazolidinediones PPARγ agonists failed to rescue cells from RSL3-induced ferroptosis, suggesting that thiazolidinediones might exert anti-ferroptosis effect regardless of PPARγ (Doll et al., 2017). Reportedly, these drugs can alleviate the glomerulosclerosis and tubulointerstitial fibrosis of various renal disease models (McCarthy et al., 2000; Chung et al., 2005; Panchapakesan et al., 2005; Omasu et al., 2007; Kawai et al., 2009; Han et al., 2010; Bilan et al., 2011; Koch et al., 2012; Shui et al., 2017; Sun et al., 2017; Németh et al., 2019; Sun et al., 2019; Wei et al., 2020), and ameliorate chronic renal allograft dysfunction (Kiss et al., 2010; Deng et al., 2019).

#### 4.1.5 DPP4 Inhibitors

In the absence of TP53, ferroptosis can be inhibited by downregulation of DPP4 or administration of DPP4 inhibitors (e.g., alogliptin, linagliptin, and vildagliptin) (Xie et al., 2017). Additionally, DPP4 represents a precise druggable target in CKD. These DPP4 inhibitors were proved to exhibit renoprotective effects and ameliorate the glomerulosclerosis and tubulointerstitial fibrosis in different diseases (Kanasaki et al., 2014; Vavrinec et al., 2014; Tanaka et al., 2016; Tsuprykov et al., 2016; Uchida et al., 2017; Oraby et al., 2019; Pengrattanachot et al., 2020; Tang et al., 2021).

#### 4.1.6 Others

Recent studies have demonstrated that liproxstatin-1 attenuates renal fibrosis induced by UUO through inhibiting ferroptosis in renal tubular epithelial cells (Zhang et al., 2021). Ferrostatin-1 is a widely used ferroptosis blocker, which exhibits renoprotective effects in obesity-induced renal fibrosis, inflammatory cell infiltration by inhibiting ferroptosis in high-fat diet mice (Li et al., 2022). It might reverse pathological parameters and improve renal function of folic acid-induced AKI mice, which is a model for studying nephrotoxic tubule damage and gradual progression of renal fibrosis (Li X. et al., 2020). Puerarin has been demonstrated to protect against heart failure via mitigating ferroptosis (Liu et al., 2018), which also ameliorates renal fibrosis by reducing oxidative stress induced-epithelial cell apoptosis via MAPK signal pathways (Zhou et al., 2017). Baicalein restrains ferroptosis by abrogating ALOX-mediated lipid peroxidation (Xie et al., 2016b; Yang et al., 2016), and can inhibit renal fibrosis in a variety of ways (Wang W. et al., 2015; Wang et al., 2016; Hu et al., 2017). U0126, the ERK inhibitor, was proved to protect against cell death induced by erastin in MEFs (Gao et al., 2015). It has also been reported to ameliorate renal fibrosis in different renal proximal tubular epithelial cells (Chen et al., 2014; Weng et al., 2020). FG-4592 pretreatment plays a protective role at the early stage of folic acid-induced renal fibrosis and other kidney injury through alleviating ferroptosis (Li X. et al., 2020) However, in the UUO mice model, even high dose treatment of FG-4592 has little effect on renal fibrosis (Kabei et al., 2020).

### 4.2 Ferroptosis Inducers

Interventions preventing cell death could initially prevent parenchymal cell loss thus protecting the kidney early in the course of kidney damage, but they may also facilitate fibrosis by blocking the downregulation of fibroblast numbers later in the course of the renal disease. EMT, charactered by loss of E-cadherin, can facilitate ferroptosis, and upregulate transcription factors, including SNAI1, ZEB1, and, TWIST1, inducing EMT-mediated tumor metastasis, elevates ferroptosis sensitivity in turn (Wu et al., 2019), while E-cadherin-mediated cell-cell contacts has been demonstrated to protect against ferroptosis (Yang W.-H. et al., 2019; Wenz et al., 2019; Wu et al., 2019). Furthermore, human cancer cell lines and organoids with a highly mesenchymal-like cell state are selectively vulnerable to ferroptosis (Viswanathan et al., 2017). It is speculated that EMT might confer susceptibility to ferroptosis-based therapies in cancers (Chen et al., 2021). Analogizing to cancer cells, it is possible that induction of ferroptosis could also attenuate renal fibrosis by selectively killing mesenchymal-like cells transited from epithelium and endothelium.

Interestingly, some ferroptosis inducers (e.g., Acetaminophen, artemisinin and its derivatives, sorafenib, sulfasalazine, and withaferin A) have also been demonstrated to alleviate renal fibrosis in various models, suggesting a complex relationship between ferroptosis and renal fibrosis (Table 4). Reportedly, sorafenib, an xCT inhibitor, can inhibit the TGF-β/Smad3-induced EMT signaling (Jia et al., 2015) and suppress the CXCR3/CXCL11-mediated macrophage infiltration (Ma et al., 2016), ameliorating renal fibrosis. Besides, an xCT inhibitor-sulfasalazine also play a protective role against renal fibrosis in the UUO rats, with decreased level of TGF-β1, ROS, and lipid peroxides (Demirbilek et al., 2007). Acetaminophen, a resistance indicator of fibrosis, and a reduction of tubular EMT in glomerular sclerosis may prevent renal oxidative stress in an obese Zucker mouse model (Wang et al., 2013; Lőrincz et al., 2015), and exerts renoprotective effects (Wang C. et al., 2015). In different models, artemisinin and its derivatives have been reported to induce ferroptosis by triggering ROS, promoting ferritin degradation, and regulating xCT/GPX4 axis (Louandre et al., 2013; Louandre et al., 2015; Sun et al., 2016), thus alleviating renal fibrosis (Cao et al., 2016; Zhang et al., 2017; Wen et al., 2019; Zhang et al., 2019; Liu et al., 2020). Withaferin A can down-regulate pro-fibrotic factors, inflammatory signaling molecules, and ER stress-related molecules and improve kidney function by decreasing uric acid (Chen et al., 2020; Zhao et al., 2021), it could also induce ferroptosis via the NRF2 pathway by targeting KEAP1 or inactivating GPX4 (Hassannia et al., 2018).

**TABLE 4:** Evidence of ferroptosis inducers alleviates renal fibrosis *in vitro* and *in vivo*.

Agents	Dosage	Model	Effects	References
Acetaminophen	30 mg/kg/day via drinking water for 26 weeks	Obese Zucker rat model	Prevents tubulointerstitial fibrosis and glomerulosclerosis by alleviating tubular EMT and inhibiting renal oxidative stress	Wang et al. (2013)
	3 μM or 10 μM	Human renal mesangial cells (HRMCs)	Exerts renoprotective effects by diminishing renal oxidative stress and p38/MAPK hyper-phosphorylation	Wang C et al. (2015)
Artemisinin	100 mg/kg/day for 16 weeks	Rats with 5/6 nephrectomy	Attenuates tubulointerstitial inflammation and fibrosis via the NF-kappaB/NLRP3 pathway	Wen et al. (2019)
	25, 50, 100 µM	Ang II-induced HK2	Attenuates tubulointerstitial inflammation and fibrosis via the NF-kappaB/NLRP3 pathway	Wen et al. (2019)
Artesunate	30 or 60 mg/kg/day for 2 weeks	UUO rats	Attenuates renal fibrosis with the up-regulation of BMP-7 and down-regulation of USAG-1	Cao et al. (2016)
	0.01, 0.1, and 1 μg/ml	TGF-β1-induced NRK-52E cells	Ameliorates TGF-β1-induced renal interstitial fibrosis by inhibition of EMT.	Zhang et al. (2017)
Dihydroartemisinin	40 mg/kg/day for 2 weeks	UUO mice	Attenuates renal fibrosis through regulation of fibroblast proliferation and differentiation via PI3K/AKT pathway and MALAT1/miR-145/FAK axis	Zhang et al. (2019); Liu et al. (2020)
	10 µM	Primary human kidney fibroblasts/HK2 cells	Attenuates renal fibrosis through regulation of fibroblast proliferation and differentiation via PI3K/AKT pathway and MALAT1/miR-145/FAK axis	Zhang et al. (2019); Liu et al. (2020)
Sorafenib	20, 40, 80 mg/kg/day, for 14 or 21 days	UUO rats	Ameliorates renal fibrosis by inhibiting TGF-β/Smad3-induced EMT signaling	Jia et al. (2015)
	5 mg/kg/day, for 10 days	UUO mice	Ameliorates renal fibrosis by inhibition of macrophage infiltration via the CXCR3/CXCL11 pathway	Ma et al. (2016)
	1, 5, and 10 μM	NRK-52E cells	Ameliorates renal fibrosis by inhibiting TGF-β/Smad3-induced EMT signaling	Jia et al. (2015)
Sulfasalazine	100 mg/kg/day, for 7 days	UUO rats	Protects against the renal interstitial inflammation and tissue damage	Demirbilek et al. (2007)
Withaferin A	3 mg/kg/day, for 14 days	UUO mice	Reverses the increases in the protein levels of pro-fibrotic factors, inflammatory signaling molecules, and ER stress-related molecules	Chen et al. (2020)
	0.1, 0.5, and 1 μM	UA-induced NRK-52E cells	Improves kidney function by decreasing uric acid via regulation of xanthine oxidase and transporter genes in renal tubular cells	Zhao et al. (2021)
	3, 5, 10 mg/kg/day, for 7 days	Hypouricemic mouse model	Improves kidney function by decreasing uric acid via regulation of xanthine oxidase and transporter genes in renal tubular cells	Zhao et al. (2021)

Besides, erastin and sorafenib could induce ferroptosis in hepatic stellate cells and are potential drugs for the treatment of hepatic fibrosis (Zhang et al., 2018). Neratinib, a demonstrated ferroptosis inducer in breast cancer (Nagpal et al., 2019), has also been shown to inhibit liver fibrosis (Park et al., 2020). Fluvastatin suppressed interstitial renal fibrosis and oxidative stress in UUO mice model (Moriyama et al., 2001) and NRK-49F cells (Ikeuchi et al., 2004). Overall, these highlight the possibility of inducing ferroptosis to achieve remission of renal fibrosis.

## 5 Other Programmed Cell Death and Ferroptosis in Renal Fibrosis

It is considered that ferroptosis interacts extensively with other modes of cell death, and even distinct from other types of programmed cell death (PCD). Multiple types of PCD have been discovered and well-studied, including apoptosis, necroptosis, pyroptosis and ferroptosis, which we would like to briefly overview their relationship with renal fibrosis.

Apoptosis is regarded as a mechanism of self-protection that eliminates dead or damaged cells that form during the physiological process of aging, and is the best-known form of PCD which was first described in 1972 (Argüelles et al., 2019). Inhibition of apoptosis has been shown to reduce kidney fibrosis (Tanaka et al., 2003; Portilla, 2014), and a number of molecules or drugs have been reported to protect the kidneys by inhibiting apoptosis (Tanaka et al., 2005).

Necroptosis, a mode of cell death dependent on the activity of RIPK1 and RIPK3 kinases, which is characterized by permeable and rupture of cell membranes can also trigger necroinflammation and release of DAMPs (Belavgeni et al., 2020). Inhibiting necroptosis was demonstrated to attenuate different type of renal fibrosis (Zhu et al., 2015; Zhu et al., 2016; Xiao et al., 2017; Dai et al., 2020; Huang et al., 2020).

Pyroptosis is caused by the cleavage of Gasdermins by various caspases, and is subsequentially executed by the insertion of the N-terminal fragment of cleaved Gasdermins into the plasma membrane, facilitate plasma membrane rupture and the release of pro-inflammatory products, which has been considered as a key fibrotic mechanism in the kidney pathology development (Kesavardhana et al., 2020; Cuevas and Pelegrín, 2021). It is a form of lytic programmed cell death initiated by inflammasomes, and HMGB1 released from pyroptotic renal tubular cells amplified inflammatory responses, which recruits immune cells to promote tissue healing and contributes to renal fibrogenesis (Raupach et al., 2006; Li Y. et al., 2021). GSDME-mediated pyroptosis is responsible for renal tubule injury and subsequentially fibrosis induced by ureteral obstruction, 5/6 nephrectomy, and DN (Li W. et al., 2021; Li Y. et al., 2021; Wu et al., 2021). For more details, interested readers may refer to these excellent recently published reviews.

In general, apoptosis is a non-lytic form of cell death and is described as an active programmed cell division process to avoid triggering inflammation to remain immunologically silent, which may confine the contribution of subsequently renal fibrosis (Fink and Cookson, 2005). However, ferroptosis, pyroptosis, and necroptosis are all considered as lytic cell death, which can contribute to the release of several immune-stimulatory DAMPs, that trigger immune responses (Tang et al., 2020). Different lytic cell deaths serve distinct purposes, determine different profiles of the inflammatory infiltrate and thus determine the association between cell death and long-term renal fibrosis (Belavgeni et al., 2020).

The critical role of necroptotic pathway lies in the host response to pathogen infection, which is triggered when apoptosis is hindered (Upton et al., 2010; Brault and Oberst, 2017). Pyroptosis is a primary cellular response following the sensing of pathogens as well as danger signals, which mainly includes DAMPs, pathogen-associated molecular patterns (PAMPs). The most significant initiator of pyroptosis is NLRP3 inflammasome which can be activated by various stimuli (Jo et al., 2016). However, as mentioned above, a key feature of ferroptosis is intracellular overload iron and catalysis of lipid peroxidation, which are both common and worsen in CKD with disease progression (Naito et al., 2015). In addition, proactive high-dose iron supplementation due to renal anemia also led to renal iron toxicity (Tanaka and Tanaka, 2015; van Raaij et al., 2018b). Thus, diverse chronic kidney disease creates an appropriate condition for the occurrence of ferroptosis. Although these different types of PCD have multiple functions in various physiological states and in a host’s response to external damage, there is clear evidence of crosstalk or interactions (Wang and Kanneganti, 2021).

## 6 Conclusion and Perspective

We have witnessed an outbreak of ferroptosis research in biomedicine over the past decades. Generally, it is a form of iron-dependent, lipid peroxidation-mediated regulated necrosis, which is strictly under controlled. This type of cell death may play a fairly important role in a wider range of physiological and pathophysiological processes (Qiu et al., 2020). On the other hand, a variety of studies on ferroptosis have detected only a few non-specific indicators, such as glutathione (GSH) levels and GPX4 levels, to demonstrate the presence of ferroptosis, which may possibly exaggerate the role of ferroptosis to some extent due to the variety of oxidative stress.

Despite a large number of recent studies have implicated hat ferroptosis is related to renal fibrosis, its complex and finely concrete mechanism is still far from being resolved. More research is warrant to elucidate whether and how pharmaceuticals or chemicals mentioned in this article affect renal fibrosis by regulating ferroptosis.
